# Supplementary material for: Canada First Nations Strengths in Community-Based Primary Healthcare
Source: Int J Environ Res Public Health. 2022 Oct 19;19(20):13532. doi: 10.3390/ijerph192013532 (PMC9602454; doi:10.3390/ijerph192013532)
Supplement: Supplementary file 1 [file ijerph-19-13532-s001.zip › ijerph-1904872-supplementary.pdf]

Supplemental Table S1. Consolidated criteria for reporting qualitative studies (COREQ): 32-item checklist.

No	Item	Guide questions/description
Domain 1: Research team & reflexivity		
Personal Characteristics		
1.	Interviewer/facilitator	Which author/s conducted the interview or focus groups?<br>***Grace Kyoon-Achan***<br>***Rachel Eni***<br>***Wanda Phillips-Beck***<br>***Local Research Assistants***
2.	Credentials	What were the researchers' credentials?<br>***GKA, PhD***<br>***RE, PhD***<br>***WPB, PhD***<br>***Local Research Assistants, Community Affiliation***
3.	Occupation	What was their occupation at the time of the study?<br>***GKA – Associate Researcher***<br>***RE – Associate Researcher***<br>***WPB – Project Coordinator***<br>***Local research assistants – community membership***
4.	Gender	***GKA, Female***<br>***RE, Female***<br>***WPB, Female***<br>***Local Research Assistants, represented all genders (male, female, 2-spirit, non-binary)***
5.	Experience & Training	What experience or training did the researcher have?<br>***All researchers listed above had more than 10 years qualitative research experience in social sciences and community health sciences at the time of data collection for this study. The local researcher assistants had a lifetime of experience living in the communities and interacting with healthcare.***
Relationship with Participants		
6.	Relationship established	Was a relationship established prior to study commencement?<br>***Yes, we visited all communities and presented the project to community leadership. We received written consent to conduct the project through Band Council Resolutions.***
7.	Participant knowledge of the interviewer	What did the participants know about the researchers? E.g., reasons for doing the research?<br>***Reasons for doing the research were described prior to the interviews, i.e., collection of data from the community-level***

		to understanding strengths for primary health care transformation.
8.	Interviewer Characteristics	What characteristics were reported about the interviewer/facilitators? E.g., Bias, assumptions, reasons and interests in the research topic<br>***Acknowledgement of the researcher affiliations with the University of Manitoba and First Nations Health & Social Secretariat of Manitoba or community membership.***
9.	Methodological Orientation & Theory	What methodological orientation was stated to underpin the study?<br>***Grounded Theory and Community-based Participatory Research***
Participant Selection		
10.	Sampling	How were participants selected?<br>***Convenience to purposive (snowballing) and then theoretically sampling sufficiency***
11.	Method of Approach	How were the participants approached?<br>***Face-to-face***
12.	Sample Size	How many participants were in the study?<br>***180 individual interviews and 5 focus groups***
13.	Non-participation	How many people refused to participate or dropped out? Reasons?<br>***There was zero refusal as all participants wanted their voices heard on the issues being discussed***
14.	Setting of data collection	Where was the data collected?<br>***All the data was collected in the communities, at homes, homecare settings, health centres, nursing stations or at the school. The focus groups were held at health centres or community halls.***
15.	Presence of non-participants	Was anyone else present besides the participants and researchers? ***No***
16.	Description of sample	What are important characteristics of the sample? E.g., demographic data, date<br>***First Nation community members, utilizers of healthcare in the communities. Some spoke their Indigenous languages fluently. Helped to elaborate on meaning from community perspectives.***
Data collection		
17.	Interview Guide	***Were questions, prompts, guides provided by the authors? Was it pilot tested? The interview guide was developed iteratively by the authors with input from community partners. Our interview guide evolved alongside our iterative analysis. This meant that as certain themes reached sufficiency, we modified our prompts to explore discrepancies***

		to further enrich the analysis. Yes, the interview guide was pilot tested.
18.	Repeat interviews	Were repeat interviews carried out? If yes, how many? ***We did not repeat any interviews.***
19.	Audio/visual recording	Did the research use audio or video recording to collect the data? ***All interviews were audio-recorded, transcribed verbatim, and de-identified prior to analysis.***
20.	Field notes	Were field notes made during and/or after the interview or focus groups? Yes
21.	Duration	What was the duration of interviews and focus groups? ***Interviews were from 30-90 minutes in duration, typically about one hour. Focus groups were 6 plus hours in duration.***
22.	Data Saturation	Was data saturation discussed? ***In the methods section, we describe how our sampling continued until saturation was reached***
23.	Transcripts returned	Were transcripts returned to participants for comment and/or correction? ***Yes, we returned the transcripts, and participants contributed back in focus groups to elaborate on issues they wanted to present.***
Domain 3: analysis and findings		
Data Analysis		
24.	Number of Data Coders	How many data coders coded the data? ***Two, GKA & RE***
25.	Description of the coding tree	Did authors provide a description of the coding tree? ***Yes, we coded the data by community and then by question***
26.	Derivation of themes	Were themes identified in advance or derived from the data? ***Themes were derived from the data***
27.	Software	What software, if applicable was used to manage the data? ***Nvivo 10***
28.	Participant Checking	Did participants provide feedback on the findings? ***Yes, themes and their descriptors as well as representative quotes were presented to participants for review and to ensure experiences were reflected accordingly.***
29.	Quotations presented	Were participant quotations presented to illustrate the themes/findings? Was each quotation identified? E.g., participant number ***Participant quotations are provided in the results to illustrate themes/findings for each question and identified by anonymized participant and community numbers***
30.	Data & findings consistent	Was there consistency between the data presented and the findings? ***Yes, there was consistency between data presented to and back from communities and findings as they are written***

31.	Clarity of major themes	Were major themes clearly presented in the findings? ***Yes. Our results present the major themes, which are presented, organized and discussed in the paper***
32.	Clarity of minor themes	Is there a description of diverse cases or discussion of minor themes? Yes, minor themes are discussed in the paper
